# Planning for Maternity Waiting Home Bed Capacity: Lessons from Rural Zambia

**DOI:** 10.5334/aogh.3691

**Published:** 2022-05-24

**Authors:** Taryn Vian, Jeanette L. Kaiser, Thandiwe Ngoma, Allison Juntunen, Kaluba K. Mataka, Misheck Bwalya, Viviane I. R. Sakanga, Peter C. Rockers, Davidson H. Hamer, Godfrey Biemba, Nancy A. Scott

**Affiliations:** 1 School of Nursing and Health Professions, University of San Francisco, San Francisco, CA, US; 2 WHO Collaborating Centre for Governance, Accountability, and Transparency in the Pharmaceutical Sector, University of Toronto, US; 3 Department of Global Health, Boston University School of Public Health, Boston, MA, US; 4 Department of Research, Right to Care Zambia, Lusaka, ZM; 5 Zenysis Technologies, Lusaka, ZM; 6 Mothers2mothers (m2m), Lusaka, ZM; 7 Amref Health Africa, Lusaka, ZM; 8 Department of Global Health, Boston University School of Public Health Section of Infectious Diseases, Department of Medicine, Boston University School of Medicine, Boston, MA, US; 9 National Health Research Authority, Pediatric Centre of Excellence, Lusaka, ZM

**Keywords:** mothers’ shelters, maternal health, facility delivery, occupancy rate, average length of stay, health system strengthening

## Abstract

**Background:**

Maternity waiting homes (MWH) allow pregnant women to stay in a residential facility close to a health center while awaiting delivery. This approach can improve health outcomes for women and children. Health planners need to consider many factors in deciding the number of beds needed for an MWH.

**Objective:**

The objective of the study is to review experience in Zambia in planning and implementing MWHs, and consider lessons learned in determining optimal capacity.

**Methods:**

We conducted a study of 10 newly built MWH in Zambia over 12 months. For this case study analysis, data on beds, service volume, and catchment area population were examined, including women staying at the homes, bed occupancy, and average length of stay. We analyzed bed occupancy by location and health facility catchment area size, and categorized occupancy by month from very low to very high.

**Findings:**

Most study sites were rural, with 3 of the 10 study sites rural-remote. Four sites served small catchment areas (<9 000), 3 had medium (9 000–11 000), and 3 had large (>11 000) size populations. Annual occupancy was variable among the sites, ranging from 13% (a medium rural site) to 151% (a large rural-remote site). Occupancy higher than 100% was accommodated by repurposing the MWH postnatal beds and using extra mattresses. Most sites had between 26–69% annual occupancy, but monthly occupancy was highly variable for reasons that seem unrelated to catchment area size, rural or rural-remote location.

**Conclusion:**

Planning for MWH capacity is difficult due to high variability. Our analysis suggests planners should try to gather actual recent monthly birth data and estimate capacity using the highest expected utilization months, anticipating that facility-based deliveries may increase with introduction of a MWH. Further research is needed to document and share data on MWH operations, including utilization statistics like number of beds, mattresses, occupancy rates and average length of stay.

## Background

Maternal and neonatal disorders are responsible for over two million deaths globally each year, with sub-Saharan Africa accounting for 65% of all maternal deaths due to pregnancy-related complications [1,2]. Lack of access to skilled delivery personnel and services, especially for women who live in remote rural areas, contributes to high rates of maternal mortality. Timely access to emergency obstetrical care can avoid up to 98% of maternal mortality in sub-Saharan Africa [3].

Maternity waiting homes (MWH) allow pregnant women to stay in a residential facility close to a health center while awaiting delivery [4]. Using an MWH can lead to improved health outcomes for women and children. Lower rates of maternal and perinatal death were reported from communities with MWHs in Liberia [5]. Maternal deaths and stillbirth rates in Ethiopia were significantly lower among women who used MWHs compared to women who did not [6,7]. In Zambia, improved MWHs were associated with 1.67 increased odds of facility delivery, as well as increased odds of postnatal attendance, counselling for family planning and breastfeeding, and receiving parenteral antibiotics, blood transfusion, and caesarean section [8].

Designing MWHs requires formative assessment to assure acceptable design. Improperly designed homes may offer culturally inappropriate services or lack sufficient linkages with the health system, hampering utilization. Research indicates that MWHs are less likely to be used if perceived as too small, crowded or unsafe, lacking privacy, and having poor hygiene and facilities, inadequate supplies of water and firewood, and lack of supervision by health staff [9,10,11].

One essential component of MWH design is bed capacity, that is, the maximum number of beds which can be installed or set up at any given time for use by pregnant women. Yet, predicting needed bed capacity in any health setting is complicated, even in high income countries with good access to data [12,13]. Health planners need to consider many factors in deciding the optimal number of beds needed for a facility. Volume is the indicator most used to guide bed size and distribution decisions [14], but use is variable [15] and maximum capacity is limited by the number of people who can reach and use a facility [16]. Assuring access to health services in rural and remote areas is especially challenging because population density is low [17].

Predicting bed need for MWHs is complicated by the difficulty of assessing gestational age in low-resource settings due to lack of ultrasound technology and late presentation at first antenatal visit [18]. Women may not know their due date, complicating the prediction of average length of stay in the MWH. In Ethiopia, one study found that 60% of women were admitted within 24 hours of the birth, 25% stayed 1–7 days, and 16% stayed over 7 days [19], while another study estimated average length of stay at 20 days [6]. Inadequate estimates of capacity can affect operating costs as well as access for pregnant women. Too few MWH beds and women are unable to benefit from the MWH; yet, too many beds may increase costs and lower efficiency.

## Objective

The objective of this case study is to describe how capacity for MWHs was estimated in rural Zambia prior to the construction of ten MWH in Eastern and Southern Provinces as part of a dual impact and implementation evaluation [20,21]. We then use MWH occupancy rates to assess actual use of facilities over a 12-month period in comparison to the built capacity, and discuss lessons learned for MWH planning in other settings.

## Methods

### Study Setting

MWH were built in 10 randomly selected rural health center (RHC) sites in the rural districts of Choma, Kalomo, and Pemba of Southern province; and Nyimba district of Eastern Province. Population density in Southern Province is 18.6 persons per sq km, while in Nyimba district of Eastern Province, population density is 8.1 persons per sq km [22]. The RHC catchment area population sizes ranged from 5 000 to 11 000, with an average of 49 villages in each catchment area. The RHCs were situated between 10 and 135 km from their district urban centers. The roads and transport options between the sites were challenging, often isolating the community groups involved in the intervention. Further information on the study site eligibility criteria and random selection process is provided elsewhere [20].

Barriers to facility access among this population include distance to health facility, transportation challenges, lack of financial resources, and sociocultural factors such as spouses and older family members who can influence women’s decisions related to care-seeking [23,24]. Difficult geography, lack of available transport options, and weather can increase travel time and exacerbate barriers [25]. Travel on the mostly packed dirt roads is more difficult in the rainy season, which is roughly November to April in Zambia. Opportunity costs from time lost from planting activities (Nov-Dec), and inconsistent revenue dependent on harvest season (April-Jun) pose additional barriers in these mainly agricultural districts [26].

### Intervention Description

We conducted formative research in the catchment areas of four randomly selected RHCs with existing MWHs to inform the design of the new MWHs [27]. We interviewed a sample of women who were pregnant or had a child under the age of two, men with a child under the age of two, community elders, and health staff in the four catchment areas. Based on these findings, we designed a Core MWH Model to be located close to a RHC, and that included features that enhance cultural acceptability and make women feel safe and comfortable [20]. For example, the formative research revealed that communities preferred separate sleeping areas for pregnant women versus women who had already delivered. Our building plans incorporated this feature.

The Core MWH Model included improved infrastructure and amenities such as concrete walls, improved flooring and roofs, lighting, latrines, access to water and cooking space [20]. The facilities were equipped with lockable doors and cabinets, beds, mattresses (including extra mattresses for companions accompanying the pregnant women), bedding, mosquito nets, and cooking equipment. The Core MWH Model was designed to be community-governed and operated, with policies and procedures to ensure smooth operations and transparent financial management.

In calculating the bed capacity for the Core MWH Model, our goal was to estimate a building size that could handle demand during the busiest months. Cost and logistical constraints meant that we could only use one size building, so we needed to consider data across the 10 sites.

We used data from 2012 to 2014 for planning, relying on publicly available government information, data from other studies, and our own formative research data. We estimated expected demand in two ways. First, we estimated births per year in each RHC’s catchment area using data on total catchment area population in 2012, applying the rural crude birth rate from the 2013–2014 Demographic and Health Survey [28]. Secondly, we gathered data on actual births per month in 2014 at each RHC from the Zambian health management information system (HMIS). Based on our formative research study, we estimated that the average length of stay for pregnant mothers would be about 10 days [27].

In 2014, about 42% of Zambian women nationwide delivered at home [28], and the odds of a woman delivering in a facility in rural areas were lower as distance to the facility increased [27]. We expected the Core MWH Model would be attractive, and conservatively planned bed capacity assuming that all women giving birth would use the facility.

We based plans on 14 beds (10 prenatal and 4 postnatal). Policies were set to account for possible over-demand, considering that if demand was very high, the postnatal beds could be repurposed for prenatal use, and 14 mattresses primarily planned for companions could accommodate the overflow. If anyone had to be turned away, the policies set criteria to favor admitting women who lived most remotely or had features of a high-risk pregnancy.

Planning for the MWH began in 2015 with construction in 2016. Most of the MWHs began operating in September and October 2016, with one MWH opening in March 2017.

### Study Design

The study design is a case study of the implementation of a project to build and operate 10 MWH in Zambia. A case study provides descriptive information about factors and context affecting implementation. These are used to develop hypotheses of how factors may influence outcomes, and under what circumstances these effects are likely to occur [29].

We conducted a retrospective review of project documents, government records, and MWH register data to explain decision-making around bed capacity for the MWHs, and to assess how planned capacity compared to actual bed use during a year of operation.

### Data Collection Methods

In each site, project staff trained the individual or individuals in charge of MWH day-to-day management. The training included how to complete the register for women staying at the MWH, recording date of admission and discharge. Project staff extracted data monthly to calculate occupancy and length of stay by month. Project staff retrospectively extracted relevant data from the 2012 Health Facility list in Zambia to obtain health facility catchment area (HFCA) population sizes.

### Variables

#### RHC location category

Rurality and remoteness are defined by environmental parameters affecting access, such as physical remoteness or population density. Zambia defines rural areas generally as having population less than 2 000, low population density, and limited roads or utilities [30]. Zambia’s Central Statistics Office does not have a definition of remote. In Canada, rural remote is defined as over 80 kilometers from a major regional hospital, while in the US, a “frontier area” (synonymous to rural remote) is defined by low population density, or a distance of 72 km (45 miles) from a primary care center to the next level of care [31]. Based on natural breaks in the distance figures of each RHC to the nearest referral hospital, we defined rural as 20–49 kilometers from the RHC to the nearest hospital, and rural-remote as 50 km or more. GPS coordinates at RHCs and referral hospitals were collected by project staff. Distance data were calculated using ArcGIS® Online. Project staff then assigned each site a category based on distance from the RHC to referral hospital. The furthest RHC was 68 km from the nearest referral hospital.

#### Population size categories

HFCA population size categories were assigned by the research team based on natural breaks in the official HFCA population size figures of each rural health center. Small was under 9 000 population; medium was 9 000–11 000 population; and large was greater than 11 000 population.

#### Projected and actual births

We calculated projected births per month by multiplying the official HFCA population size in 2012 by the crude birth rate for rural areas in the 2013/2014 Zambia Demographic and Health Survey (40.3 births per 1 000 population) and dividing by 12 months. For comparison, we also include the actual number of births registered in each HFCA from 2014, as described earlier. Actual births between August 2017-July 2018 were extracted monthly from RHC delivery records, and divided by 12 to show monthly births.

#### Projected and actual users

A user was defined as a woman who registers to stay in the MWH while awaiting delivery. We excluded postnatal users. For projected users, we assumed that for all expected births within the HFCA, the mother would stay at the MWH. See estimates of projected births, above. For actual users, we gathered data from the MWH registers.

#### Projected and actual occupied bed days

A bed day was defined as a bed being used by a woman staying at the MWH one night. Projected bed days per month were based on the projected users per month times the projected average length of stay of 10 days per user. Actual occupied bed days were gathered from the MWH registers, which recorded each user’s arrival and departure date, and reason for stay (awaiting delivery). We did not include postnatal users or postnatal bed days.

#### Low, medium, and high occupancy

There is not one standard method for identifying low or high occupancy in health facilities. In a bed occupancy projection tool, the American Hospital Association uses a sliding color scale that classifies “low” as under 65%, “medium” 65%-80%, and “high” as over 80% [32]. Similarly, a Council of Europe report found that occupancy rates for curative care beds in Europe were generally in the range of 61–82%, implying that below 61% was “low” and above 82% was “high” [33]. The U.S. Health and Human Services uses categories of 0–39.9%, 40–49.9%, 50–59.9%, 60–69.9%, and 70% or above [34]. Given that rural facilities have higher occupancy variability in general, we chose to use the following categories: under 10%, 10.1–25%, 25.1–85%, 85.1–100%, over 100%. The color scheme goes from dark blue (very low) to dark red (very high).

### Analysis

We analyzed data based on projected figures and actual data.

#### MWH occupancy

Average occupancy rate is defined as occupied bed days divided by available bed days. This figure may be shown on an annual or monthly basis. The projected occupancy was calculated based on the projected occupied bed days divided by available bed days. The available bed days per year is the number of beds available for waiting mothers (10 prenatal beds) times 365 days in a year. Available bed days per month is the available bed days per year divided by 12 months. The actual occupancy was calculated based on actual occupied bed days divided by the available bed days (10 prenatal beds x 365 days in a year, also shown by month), with standard deviation.

#### Average length of stay

Average length of stay is defined as occupied bed days divided by the total number of users (women who registered to stay in the MWH). This figure is shown by month and overall for the 12 months.

### Ethics

The overarching implementation evaluation received formal ethical approval from the Boston University Institutional Review Board (IRB), the Zambian ERES IRB, and the National Health Research Authority in Zambia, the overseeing regulatory body in the country. Both IRBs waived the need to obtain informed consent for each woman entered into the MWH register.

## Results

### Site locations and size

The majority of study sites were rural, with three of the ten study sites considered rural-remote (Table 1). The catchment area of 4 sites was small (<9 000 persons); 3 were medium (9 000–11 000 persons); and 3 were large (>11 000) size.

**Table 1:** Health facility catchment area characteristics, and projected and actual births and MWH occupancy figures by study site.


RURAL HEALTH CENTER SITE	DISTRICT	DESCRIPTION OF SITE LOCATION^a^	2012 OFFICIAL HFCA POPULATION SIZE 2012^b^	HFCA POPULATION SIZE CATEGORIES^c^	2012 PROJECTED BIRTHS PER MONTH IN HFCA^d^	2014 ACTUAL BIRTHS PER MONTH AT RHC^e^	PROJECTED OCCUPANCY OF MWH^f^		2017/2018 ACTUAL BIRTHS PER MONTH AT RHC^g^	2017/2018 ACTUAL OCCUPANCY OF MWH^h^

							BASED ON 2012 PROJECTED BIRTHS IN HFCA	BASED ON 2014 ACTUAL BIRTHS AT RHC		

**Masuku**	Choma	Rural-remote	7457	Small	16	13	53%	43%	18	47%

**Mbabala**	Choma	Rural	9943	Medium	21	24	69%	79%	31	25%

**Simakutu**	Choma	Rural	4972	Small	11	26	36%	85%	23	21%

**Jembo**	Pemba	Rural-remote	9943	Medium	21	24	69%	79%	23	64%

**Chilala**	Kalomo	Rural	11009	Large	23	20	76%	66%	18	27%

**Kanchele**	Kalomo	Rural-remote	11009	Large	23	48	76%	158%	56	151%

**Mukwela**	Kalomo	Rural	8256	Small	18	13	59%	43%	15	26%

**Siachitema**	Kalomo	Rural	11009	Large	23	40	76%	132%	25	32%

**Kacholola**	Nyimba	Rural	9443	Medium	20	15	66%	49%	11	13%

**Mkopeka**	Nyimba	Rural	8373	Small	18	17	59%	56%	20	69%


**Abbreviations:** RHC = Rural health center; HFCA = Health Facility Catchment Area. **^a^** See explanation of RHC location categories in Methods section. Rural = 20–49 km; Rural-remote = 50+ km. The furthest rural health center is 68 km from the nearest referral hospital. GPS coordinates at RHCs and referral hospitals were collected by project staff. Distance data were calculated using ArcGIS® Online. **^b^** Official HFCA size data are from the 2012 List of Health Facilities in Zambia. **^c^** HFCA population size categories were assigned by the research team based on natural breaks in the official HFCA population size figures of each rural health center. Small = <9000 population; medium = 9000–11000 population = large >11000 population. **^d^** Projected by multiplying official HFCA population size in 2012 by the Crude Birth Rate (rural) in the 2013/2014 Demographic and Health Survey (40.3 births per 1000 population). **^e^** Delivery volume data are from the Zambian health management information system (HMIS) for 2014, provided by the Saving Mothers, Giving Life project. **^f^**Projected occupancy of MWH = projected bed days/available bed days per month. Projected bed days = projected users per month (births in HFCA or delivery volume) * average length of stay (10 days). Available bed days per month = (Number of beds [10] * days in a year [365])/month (12). Average length of stay was based on formative research conducted in these districts.^27^ Assumes 100% of either HFCA births or RHC deliveries will stay at the MWH. **^g^**Rural health center delivery records extracted by research team for August 2017 – July 2018. **^h^**Actual occupancy of MWH = bed days/available bed days, averaged over 12 months. Based on MWH register data, users’ arrival and departure dates, and reason for stay (awaiting delivery).

### Occupancy

Actual annual occupancy in 2017/2018 (Table 1) ranged from 13% in Kacholola (a medium rural site in Nyimba district) to 151% in Kanchele (a large rural-remote site in Kalomo district). As mentioned, the occupancy could be higher than 100% by repurposing the postnatal beds and using the companion mattresses. Three sites had low occupancy (under 25%). Most had between 26–69% occupancy.

We found no apparent trends in MWH occupancy rates by district, rural vs. rural-remote, or season (Nov-April rainy season versus other months). The largest HFCA also had the largest standard deviation, indicating higher variability in occupancy rates month to month, compared to small and medium size HFCA.

Monthly occupancy data are shown in Table 2, grouped by district. Some study sites had consistently high monthly occupancy (e.g. Kanchele with 10 months higher than 85% occupancy) or consistently low occupancy (e.g. Kacholola and Simakutu with 10 and 7 months at below 25% occupancy, respectively). Yet, other sites vacillated between very high and very low occupancies, sometimes in consecutive months (e.g. Mkopeka ranged from 16% to 121% occupancy; Mbabala and Siachitema ranged from 1% to 70–71%). Masuku (Choma District) had the highest number of months with average occupancy (nine months with occupancy between 25–85%), and no month over 100% occupancy.

**Table 2:**
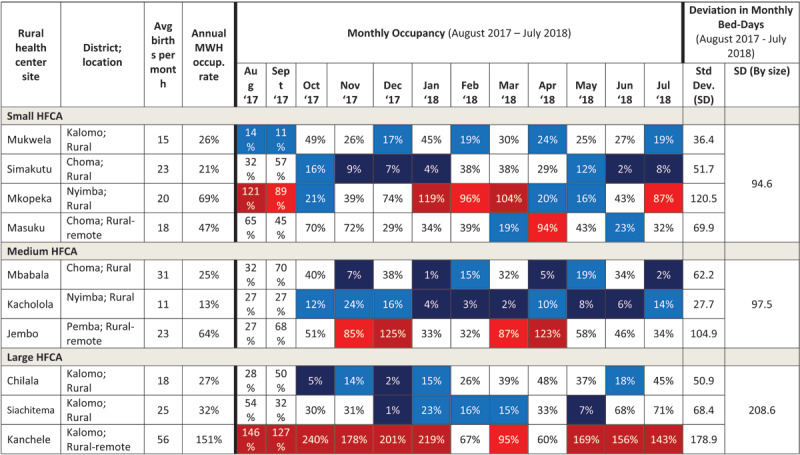
MWH monthly and annual occupancy over 12 months (August 2017–July 2018) by study site grouped by size of HFCA. *Notes:* Color coding of monthly occupancy rates: Dark Blue = very low (under 10%); Medium Blue = low (10.1–25%); White = average (25.1–85%); Medium Red = high (85.1–100%); Dark Red = very high (over 100%).

### Average length of stay

Overall average length of stay across the 10 sites was 13.4 days, ranging from 8.5 days in Simakutu to 16.8 days in Kanchele (Table 3). The months with lowest average length of stay overall were August and October (11.9 days). Average length of stay was highest in December (17.0 days).

**Table 3:** MWH monthly average length of stay in days (August 2017 – July 2018).


RURAL HEALTH CENTER SITE	AUG 2017	SEPT 2017	OCT 2017	NOV 2017	DEC 2017	JAN 2018	FEB 2018	MAR 2018	APR 2018	MAY 2018	JUN 2018	JUL 2018	OVERALL

**Choma/Pemba**													

Simakutu	5.8	10.7	6.3	6.7	4.6	5.5	19.8	16.9	9.2	7.2	2.0	3.7	8.5

Mbabala	10.2	15.8	10.5	11.5	18.0	10.5	16.4	14.8	4.7	6.5	14.2	11.6	12.2

Masuku	16.1	11.3	12.0	16.5	11.1	6.2	11.0	8.9	14.7	14.9	13.6	18.5	12.7

Jembo	7.2	16.8	10.6	16.8	23.5	15.9	11.5	14.2	19.1	25.6	10.7	8.2	14.7

**Kalomo**													

Mukwela	4.2	7.5	10.1	11.3	13.2	7.8	9.0	10.4	7.2	9.6	9.1	9.4	8.9

Chilala	17.2	15.0	2.2	14.0	5.0	11.8	10.4	11.0	16.0	10.4	9.0	14.1	12.0

Siachitema	11.1	10.7	10.2	18.6	5.7	5.9	11.8	7.8	13.9	4.2	13.5	13.0	11.0

Kanchele	14.1	16.7	18.6	16.2	19.4	17.9	11.8	16.3	11.5	19.4	18.0	17.0	16.8

**Nyimba**													

Kacholola	14.0	16.0	3.6	12.2	12.5	3.3	3.0	6.0	15.0	8.7	5.7	8.1	8.9

Mkopeka	17.1	11.0	11.8	14.3	16.5	15.2	19.5	18.4	10.4	12.7	32.5	25.7	16.0

**All Sites**													

	11.9	13.4	11.9	14.9	17.0	12.3	13.3	14.0	13.2	13.5	13.9	13.4	13.4


### Projections versus Actual

The two methods of projecting expected occupancy based on birth estimates yielded inconsistent results (Table 1). The results based on actual facility-based births in 2014 were higher in 5 sites than the estimates based on 2012 population data, but lower in the other five sites. In Simakutu, Kanchele, and Siachitema, projected occupancy based on the 2014 actual facility-based births was 85%, 158%, and 132%, respectively, given the 10-bed capacity of the Core MWH Model.

Neither method was clearly better at projecting occupancy when compared to the actual 2017/2018 occupancy rate at the MWHs. In 5 sites, the projections based on the 2014 data were a better approximation, whereas in the other 5 sites, the 2012 estimates landed closer to actual experience.

Our estimate of average length of stay, based on formative research interviews in the communities, was 10 days. This was about one-third lower than the actual average length of stay of 13.4 days.

## Discussion

The aim of this study was to glean lessons learned for planning MWH capacity in rural and remote areas by describing how MWH facility size was decided in rural Zambia, and assess whether the estimates were adequate given the actual occupancy experienced after construction of 10 MWHs. After making projections using multiple data sources, the MWH facility size was ultimately based on actual delivery data from 2014 (that is, 2 years before the MWHs opened), assumptions regarding average length of stay, and the proportion of pregnant women who would seek to use an MWH before giving birth.

We found that actual occupancy was highly variable, ranging from 13% to 151% in the sites studied. No site had occupancy in the range of 25–85% for all 12 months, though more than half the sites had occupancy in this range for half the year or more. Some sites had consistently high occupancy and some had consistently low. One site had over 100% occupancy for 9 out of 12 months, while 2 sites had occupancy below 10% for 5 out of 12 months. Average length of stay was variable as well, ranging from 8.5 to 16.8 days (average 13.4 days). Through the use of postnatal beds and extra mattresses, the Zambian MWH staff were mostly able to accommodate the excess utilization and did not have to turn women away.

Occupancy rates and actual length of stay have not been reported in many studies of MWH operations due to lack of record-keeping [35]. The Ethiopian Public Health Institute estimated that MWH occupancy was 29% of available bed capacity in 2016 [36]. Another study of one 48-bed MWH in Ethiopia reported use by 500–700 women per year, with average length of stay of 20 days [6] or about 57–80% occupancy. In Kenya, a 20-bed MWH near a hospital had very low utilization and average length of stay varying widely from 2 days to 1.5 months [37]. A study in Liberia reported MWH average length of stay of 15.9 days for women awaiting delivery [38]. This study did not report occupancy, but noted that limited capacity was a challenge.

Very high and very low occupancy for facilities can be problematic. Very high bed occupancy means that pregnant or postnatal women may have to sleep on a mattress on the floor and there will be no room for their companion. In the worst case, it might mean that some women are turned away. This may affect their access to timely obstetric services at such a late gestational age. Women would also incur costs and time having to return home while heavily pregnant. Quality of services may also be lower in very high occupancy situations. On the other hand, very low occupancy could make the women who do stay feel unsafe because they are few. It may also deter others in the community from wanting to stay at the facility [39]. At the same time, some idle capacity is beneficial because it avoids the cost of delaying or denying admission [40]. We planned for MWH size knowing that some structures were likely to have low utilization but not, we hoped, very low utilization.

We found that rural-remote MWHs in Choma, Pemba and Kalomo Districts had the highest occupancy rates, even though they were in a mix of HFCA population sizes (small, medium, and large). Though we would have anticipated HFCA population size to be highly correlated with MWH occupancy, it was not. As a rule, very large catchment areas likely should have had a larger MWH (e.g. Kanchele), while we probably could have planned for smaller MWH at smaller sites, to decrease initial costs and long-term upkeep. Yet, there is a cost to developing and implementing different designs for construction, including the difficulty of monitoring and assuring contractor accountability.

Occupancy is driven by births but likely affected by a whole range of factors that we cannot easily plan for, including opportunity costs for women due to missed work and need to arrange for care of children at home, weather, long travel times, available transportation options, and women’s perception of risk [17,36]. Studies have shown that client-side factors influence use of MWH. For example, in Ethiopia researchers found that offering meal service and adequate cooking space at the MWH, being able to decide for oneself whether to use a facility, and having someone at home to care for children affected utilization [41,42,43]. As more demographic and health information data become available in specific countries and regions, variables such as these could be input into models used to predict demand.

Our study suggests several principles to guide planning for MWH in other settings:

First, using the most recent actual data on births per month (preferably in the year prior to designing MWH) seems to provide the best planning source for anticipating bed need. Monthly birth data are more helpful than the average annual births, to understand variability. We had anticipated seasonal patterns in use but found none, yet this may not be the case in all countries. Health planners should also obtain the best information available on HFCA boundaries and any government plans for changing boundaries. For this project, the HFCA boundaries changed during our planning phase, new health facilities were opened, and villages were reassigned to different catchment areas. This required changes as we adjusted sites.

Secondly, planners should try to optimize MWH capacity around the highest possible demand. Facilities should be able to handle the busiest month or months, and be willing to accept months with low capacity in order to achieve equity goals and not have to turn anyone away. It may make sense to construct MWH in batches to be able to gather more data on actual use of facilities to estimate average length of stay and possible patterns in occupancy based on seasonality or population distribution. Where financially and operationally feasible, it is preferable to vary the facility plans to allow certain HFCAs with expected high utilization to have a MWH with a higher bed size, and very small communities to have a smaller MWH. The cost of monitoring construction with varying facility plans must be considered in making this decision.

Third, MWH facilities should have a plan to handle periodic surges in use above the bed capacity. In our sites, we provided mattresses for companions that could be repurposed to accommodate pregnant women (the companions could be asked to stay elsewhere or return home). We also experienced low demand for the postnatal beds, and were able to use these beds for pregnant women if needed (postnatal women could either stay longer in clinic beds, or return home).

Fourth, planners should consider how national policy and client-factors may affect bed capacity estimates. In Zambia, MWHs were recommended to all pregnant women approaching their estimated delivery date. In many other countries, MWHs are designed to be used only for pregnant women that are identified as at high risk for complications or women who live at a long distance from the RHC [35,44]. In such cases, bed capacity estimates would be modified. It is important to consider context and policy characteristics (e.g., risk-based, distance) related to the population the MWHs are established to serve. Data on characteristics of MWH (indicators of quality) may also be used to model expected demand.

Finally, researchers studying the implementation and effectiveness of MWH should share data on actual bed size, occupancy rates, average length of stay, and other MWH operational data. Comparative data might be helpful for countries seeking to establish MWH in areas that have not previously used them. A study of MWHs in Liberia reported on 5 new or renovated 8-bed facilities in HFCAs ranging in size from 2 998 to 22 637 population [11]. The researchers calculated costs in relation to maternal deaths in intervention versus comparison sites, but did not present data on efficiency of use (e.g. occupancy, length of stay). In Ethiopia, a study of one 8-bed MWH noted that only 24% of mothers who delivered at the primary health facility stayed in the MWH without providing occupancy or average length of stay [36]. Additional data on MWH capacity and utilization statistics would be helpful for this field of study. As more data are collected, it may be possible to develop advanced models to better forecast demand, which could help improve efficiency [45]. Future research should aim to better understand the factors that drive high monthly variability in MWH occupancy.

## Limitations

The main weakness of this study is the sample size of ten MWHs, limiting generalizability to the whole country, and the single-country setting limits international generalizability. In addition, our study was limited to one year of actual MWH data. We do not know how occupancy or average length of stay might change in the future. Yet, we believe that the details on factors and data considered when planning and the experience of the 10 sites can provide a case study with lessons for guiding planners elsewhere in Zambia and in other countries.

## Conclusion

This study indicates that it is challenging to plan for MWH capacity, especially with publicly available data. Our analysis suggests planners should try to gather actual monthly birth data from as recently as possible, and to base capacity on the highest expected utilization months, anticipating that facility-based deliveries may increase with introduction of a MWH. Further research is needed to document and share data on actual MWH operations, including utilization statistics like number of beds, mattresses, occupancy rates and average length of stay. Remote-living rural women are likely to benefit most from having access to MWH and facility-based delivery services.
